# A VSV-based Zika virus vaccine protects mice from lethal challenge

**DOI:** 10.1038/s41598-018-29401-x

**Published:** 2018-07-23

**Authors:** Jackson Emanuel, Julie Callison, Kimberly A. Dowd, Theodore C. Pierson, Heinz Feldmann, Andrea Marzi

**Affiliations:** 1 Laboratory of Virology, Division of Intramural Research, National Institute of Allergy and Infectious Diseases, National Institutes of Health, Hamilton, MT USA; 2 Laboratory of Viral Diseases, Division of Intramural Research, National Institute of Allergy and Infectious Diseases, National Institutes of Health, Bethesda, MD USA

## Abstract

Infection with Zika virus (ZIKV) is commonly mild in humans but has been associated with alarming negative health outcomes including Guillain-Barré syndrome in adults and microcephaly in fetuses. As such, developing a vaccine for ZIKV is a global public health priority. Recombinant vesicular stomatitis virus (VSV) expressing the Ebola virus (EBOV) glycoprotein (GP) has been successfully used as a vaccine platform in the past. In this study, two novel VSV-ZIKV vaccines were generated utilizing the favorable immune targeting of the existing VSV-EBOV vector. In addition to the EBOV GP, these new vaccines express the full-length pre-membrane and envelope proteins or pre-membrane and truncated soluble envelope proteins as antigens. Efficacy testing of both of the VSV vectors against ZIKV was conducted in IFNAR^−/−^ mice and resulted in uniform protection when a single dose was administered 28 days prior to lethal challenge. Furthermore, this vaccine is fast-acting and can uniformly protect mice from lethal disease when administered as late as 3 days prior to ZIKV challenge. Thus, VSV-ZIKV vectors are promising vaccine candidates and should move forward along the licensure pathway.

## Introduction

Zika virus (ZIKV) is an emerging human pathogen that has gained attention for its threat to global public health. Over the past decade, sporadic outbreaks of ZIKV occurred in Micronesia, French Polynesia, South East Asia and the Americas^1–3^. Clinical cases typically present as a mild febrile illness, including symptoms such as headache, myalgia, conjunctivitis and maculopapular rash^4,5^. ZIKV infection in pregnant women, however, has been associated with deleterious effects on fetal brain development, potentially causing miscarriage or microcephaly^6,7^. ZIKV infection in adults has also been linked to Guillain-Barré syndrome, a debilitating and potentially life-threatening condition of the peripheral nervous system^8–10^. ZIKV is primarily transmitted by *Aedes* mosquito vectors or via sexual contact^11–13^. From 2015 to 2017, there were more than 800,000 human cases of confirmed and suspected ZIKV infection in the Americas, indicative of highly rapid spread^14–16^. Even though the initial global public health emergency status has been cleared, according to the WHO, ZIKV still represents an enduring public health challenge^17^.

ZIKV is a member of the Spondweni virus clade in the *Flavivirus* genus. It shares many structural similarities with other pathogenic flaviviruses, such as dengue virus, Japanese encephalitis virus (JEV), and West Nile virus^18,19^. The ZIKV genome comprises a positive-sense, single-stranded RNA of about 11-kb in length which is expressed as a single polyprotein. This polyprotein is cleaved by viral and host proteases into 10 functional proteins^19,20^. The structure of the ZIKV particle has been solved by cryo-electron microscopy and reveals that the envelope protein (E) and the membrane protein (M) compose the outermost layer and are anchored to the host-derived lipid envelope by transmembrane domains^19,21^. ZIKV neutralizing antibodies are known to target a variety of epitopes on the E protein surface, making this protein an ideal candidate for vaccine development^22,23^. The pre-cursor membrane (prM) protein complexes with the E protein shortly after synthesis and functions to prevent premature membrane fusion during viral egress. Cleavage of prM occurs during transit of virus particles through the trans-Golgi network and is required for the formation of infectious virus particles^24^. *In vitro* expression of the full-length prM and E proteins in mammalian cells is sufficient to form non-infectious subviral particles (SVPs) that have shown to be promising antigens for flavivirus vaccines, including multiple ZIKV DNA vaccine candidates that have been evaluated in clinical trials^22,25,26^. While SVPs are structurally heterogeneous, they appear to incorporate E protein heterodimers that comprise the functional and antigenic building blocks of virions important for eliciting antibodies that bind the complex features of infectious virus particles^27^.

**Figure 1:**
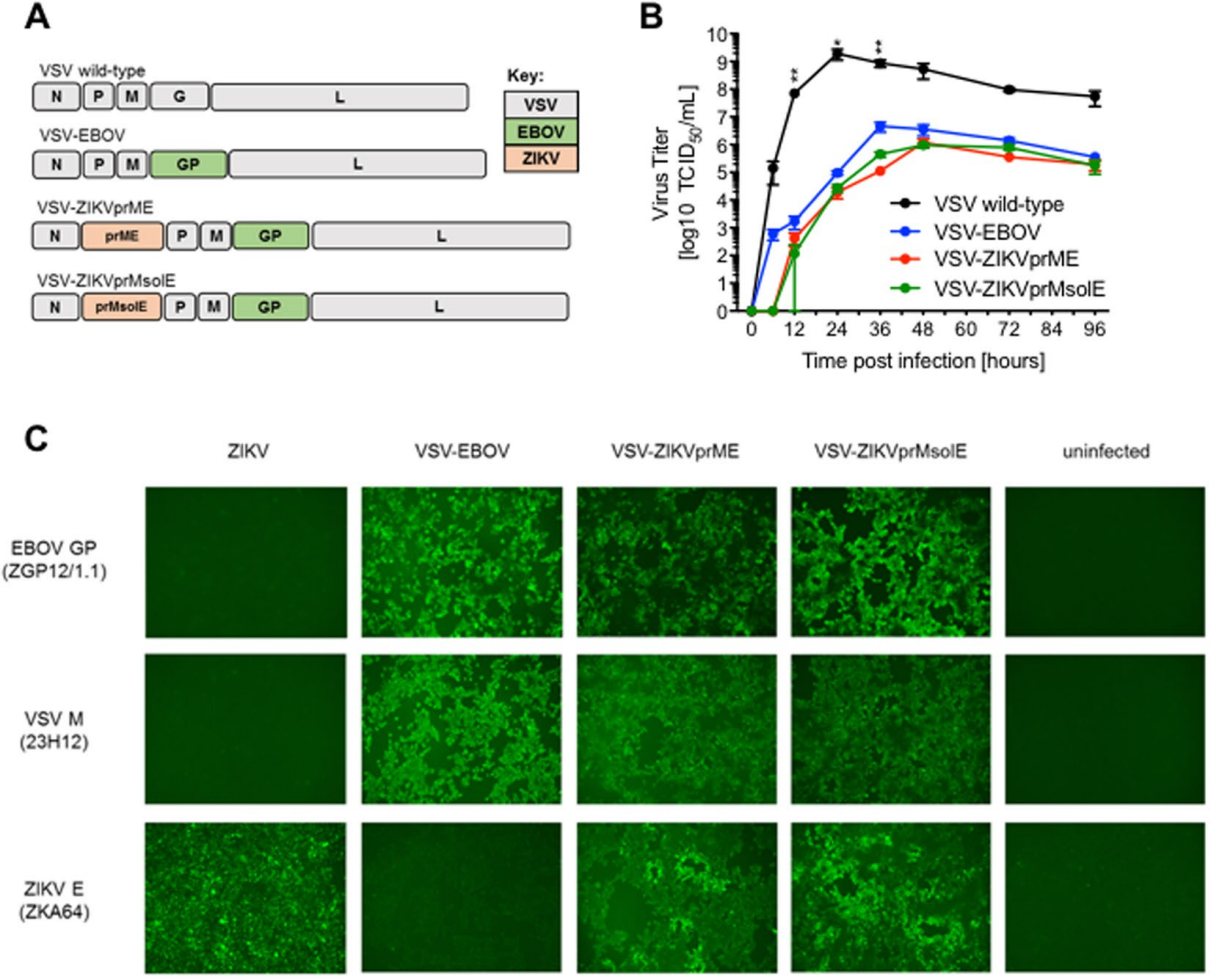
Design and *in vitro* characterization of VSV-ZIKV vaccines. (**A**) Genome organizations of the VSV-ZIKV vectors. N nucleoprotein, P phosphoprotein, M matrix protein, G glycoprotein, L polymerase, GP glycoprotein, prM pre-cursor membrane protein, E envelope glycoprotein. (**B**) *In vitro* growth kinetics of VSVs grown on VeroE6 cells. One representative experiment in triplicates is shown. Error bas indicate standard deviation. Significantly different results are indicated as follows: **p < 0.01 and *p < 0.05. (**C**) Immunofluorescence analysis confirmed the presence of the EBOV GP and ZIKV E antigens in VSV-infected VeroE6 cells.

Vesicular stomatitis virus (VSV) is a member of the *Rhabdoviridae* family. Although VSV can cause disease in livestock and other animals, it is highly restricted by the human interferon response and generally does not cause any or only very mild disease in humans^28,29^. VSV has proven to be a remarkably tractable vector, suitable for genetic manipulation. Recombinant VSVs have been previously generated for use as replication-competent vaccines^30–35^. A prominent example is the VSV-based Ebola virus (EBOV) vaccine VSV-EBOV (also known as rVSV-ZEBOV), which expresses the EBOV glycoprotein (GP) instead of the VSV glycoprotein G (Fig. 1A). This substitution attenuates the vaccine, as it replicates slower, to lower but tolerable titers, and lacks VSV G, which is the neurotropism determinant in animals^36,37^. Replacement with EBOV GP alters the virus tropism in a manner that favors a robust immune response^38^. In non-human primates it has been found that VSV-EBOV stimulation of CD4 T cells is essential for antibody-dependent protection against EBOV^39^. Past VSV-based vaccines have demonstrated rapid conveyance of immunity, suitable for deployment in outbreak scenarios and post-exposure prophylaxis^33,40^. The VSV-EBOV vaccine is currently in clinical trials with promising results^41–43^. It has also been previously demonstrated that the VSV-EBOV can be used as a vaccine platform by expressing additional vaccine antigens, while retaining its immunogenicity against EBOV^44,45^.

Previous efforts have been made at creating a VSV-based ZIKV vaccine which included the VSV G in addition to the ZIKV antigens, however, protection against lethal ZIKV challenge was only performed in the offspring of vaccinated female mice, not the vaccinated animals themselves^34^. The present study details the generation, characterization, and testing of two distinctive VSV-ZIKV vaccines, based on the VSV-EBOV vector (Fig. 1A). This strategy was chosen to take advantage of the favorable targeting of the EBOV GP to mediate entry into important immune cells^46^. Efficacy studies were performed against lethal ZIKV and EBOV challenge in mice. For ZIKV challenge, C57BL/6 interferon α receptor knock out (IFNAR^−/−^) mice were used, as ZIKV neither replicates efficiently nor causes lethal disease in wild-type mice^47–49^. Both vaccines confer 100% protection against lethal ZIKV and EBOV challenge with rapid protection against ZIKV infection, as mice vaccinated at least 3 days prior to challenge are uniformly protected from lethal disease.

## Results

### Vaccine construction and characterization

Two ZIKV vaccine vectors were generated by introducing codon-optimized sequences encoding the full-length pre-membrane and envelope proteins (prME) or pre-membrane and soluble envelope proteins as antigens (prMsolE) to the VSV-EBOV vector (Fig. 1A). As these vaccine vectors express two additional proteins encoded in their genome, we expected an attenuation in growth kinetics compared to the parental vector, VSV-EBOV. To test this hypothesis, we analyzed virus replication in triplicate on VeroE6 cells infected with an MOI of 0.01 for 96 hours. As expected, wild-type VSV grew more rapidly and to significantly higher titers (~1 × 10^9^ tissue culture infectious dose of 50% (TCID_50_)) as compared to recombinant viruses. Furthermore, the growth kinetics results suggest that VSV-ZIKVprME and VSV-ZIKVprMsolE have a slower rate of replication than VSV-EBOV, although the difference was not statistically significant. Both VSV-ZIKV vaccines reached their peak titer of ~1 × 10^6^ TCID_50_ around 48 hours post-infection, whereas VSV-EBOV reached a peak titer of ~5 × 10^6^ TCID_50_ around 36 hours post-infection (Fig. 1B). The expression of EBOV GP, VSV matrix protein (M), and ZIKV E in VSV-ZIKV infected cells was confirmed by immunofluorescence assay (Fig. 1C); Western blot analysis confirmed the presence of these proteins in the cell supernatant (Fig. S1).

As the VSV-ZIKV vectors are replicating for several cycles in a vaccinated host, we wanted to verify that the ZIKV antigen-coding sequences are stably inserted in the VSV genome. To this end the VSV-ZIKV vaccines were serially passaged on VeroE6 cells 10 times. Supernatant samples were collected and RNA was extracted at each time point. PCR amplification with primers specific for the ZIKV antigen-coding sequences was performed and showed that the ZIKV prME and ZIKV prMsolE open reading frames were present throughout all 10 passages (Fig. S2A) demonstrating antigen stability in the VSV vector. Furthermore, EBOV GP expression was confirmed by Western blot analysis in the supernatant of the infected cells suggesting stable expression of all genes in the VSV genome (Fig. S2B).

### VSV-ZIKV vaccine efficacy in the IFNAR^−/−^ mouse model

**Figure 2:**
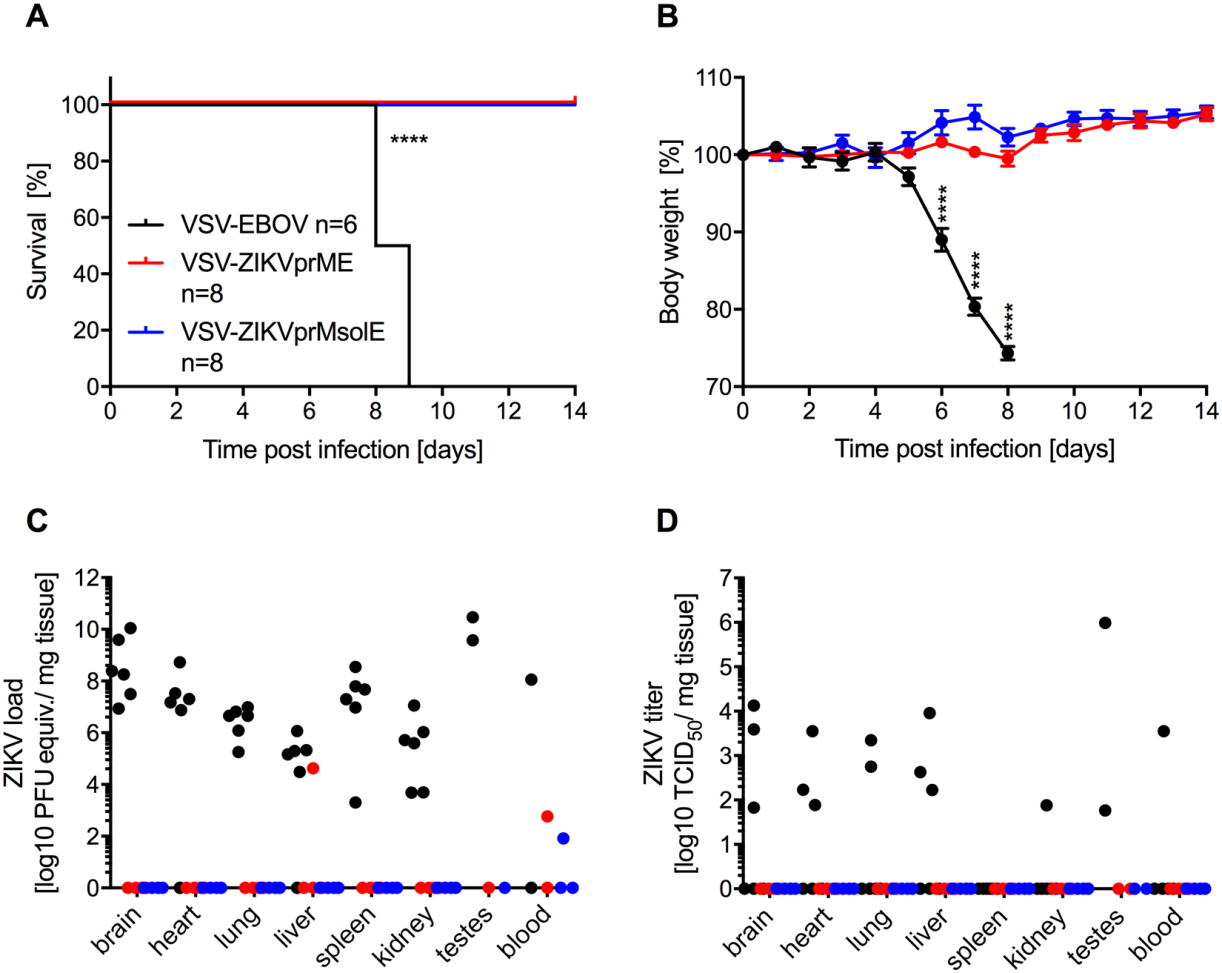
Efficacy of the VSV-ZIKV vaccines against lethal ZIKV challenge. IFNAR^−/−^ mice were IM vaccinated with a single dose of vaccine 28 days prior to lethal ZIKV challenge. (**A**) Survival curves after lethal ZIKV challenge. (**B**) Body weight changes after ZIKV challenge. On day 7 after challenge for vaccinated animals, 4 mice were euthanized for sample collection. For control animals (n = 6) samples were collected at the time when a humane endpoint was reached (days 7 and 8). (**C**) Levels of ZIKV RNA present in selected tissues. (**D**) ZIKV titers in selected tissues. Significantly different results are indicated as ****p < 0.0001.

Protective efficacy of the VSV-ZIKV vaccines was evaluated in IFNAR^−/−^ mice. Groups of 16 male and female IFNAR^−/−^ mice were intramuscularly (IM) vaccinated with 1 × 10^4^ plaque forming units (pfu) of VSV-EBOV, VSV-ZIKVprME or VSV-ZIKVprMsolE. Unexpectedly, 10 out of 16 mice in the VSV-EBOV group reached humane endpoint criteria and had to be euthanized 5–6 days post-vaccination. The 6 mice remaining in this group never developed signs of disease and were carried through the study as initially planned. Four weeks after vaccination, 12 mice per vaccine group and the 6 remaining animals in the VSV-EBOV group were intraperitoneally (IP) challenged with 1,000 LD_50_ (5,000 pfu) of ZIKV French Polynesia. Both vaccines, VSV-ZIKVprME and VSV-ZIKVprMsolE, protected 100% of the IFNAR^−/−^ mice from lethal challenge (Fig. 2A). No weight loss or signs of disease were observed in these two groups following ZIKV challenge (Fig. 2B). Conversely, the control vaccine, VSV-EBOV, failed to provide protection (Fig. 2A). All control mice (n = 6) developed signs of ZIKV disease including weight loss (Fig. 2B), ruffled fur, and neurological symptoms such as tremors, and were humanely euthanized on day 7 and 8 according to protocol. On day 7 after challenge we collected blood and tissue samples for virological analysis from 4 animals in the VSV-ZIKV vaccinated groups, and the control mice at the time of euthanasia. Gross pathology at the time of necropsy for the control mice revealed mild to severe splenomegaly and pale discoloration of the liver, whereas VSV-ZIKV vaccinated mice did not show any signs of tissue damage on day 7 post challenge. In control animals, high levels of ZIKV RNA were detected in the majority of the examined organs (Fig. 2C). By contrast, ZIKV RNA could only be detected at low levels in the VSV-ZIKV vaccinated groups, either in the blood only (VSV-ZIKVprMsolE, n = 1/4) or liver and blood from the same mouse (VSV-ZIKVprME, n = 1/4) (Fig. 2C). Virus was isolated from samples collected from all 6 control mice, with individual variation in blood, liver, heart, lung, brain, kidney, and testes (Fig. 2D). In contrast, no virus was isolated from any of the samples collected from the VSV-ZIKV vaccinated animals demonstrating equally robust protection by both VSV-ZIKV vaccines.

### VSV-ZIKV vaccine-mediated antibody responses in IFNAR^−/−^ mice

**Figure 3:**
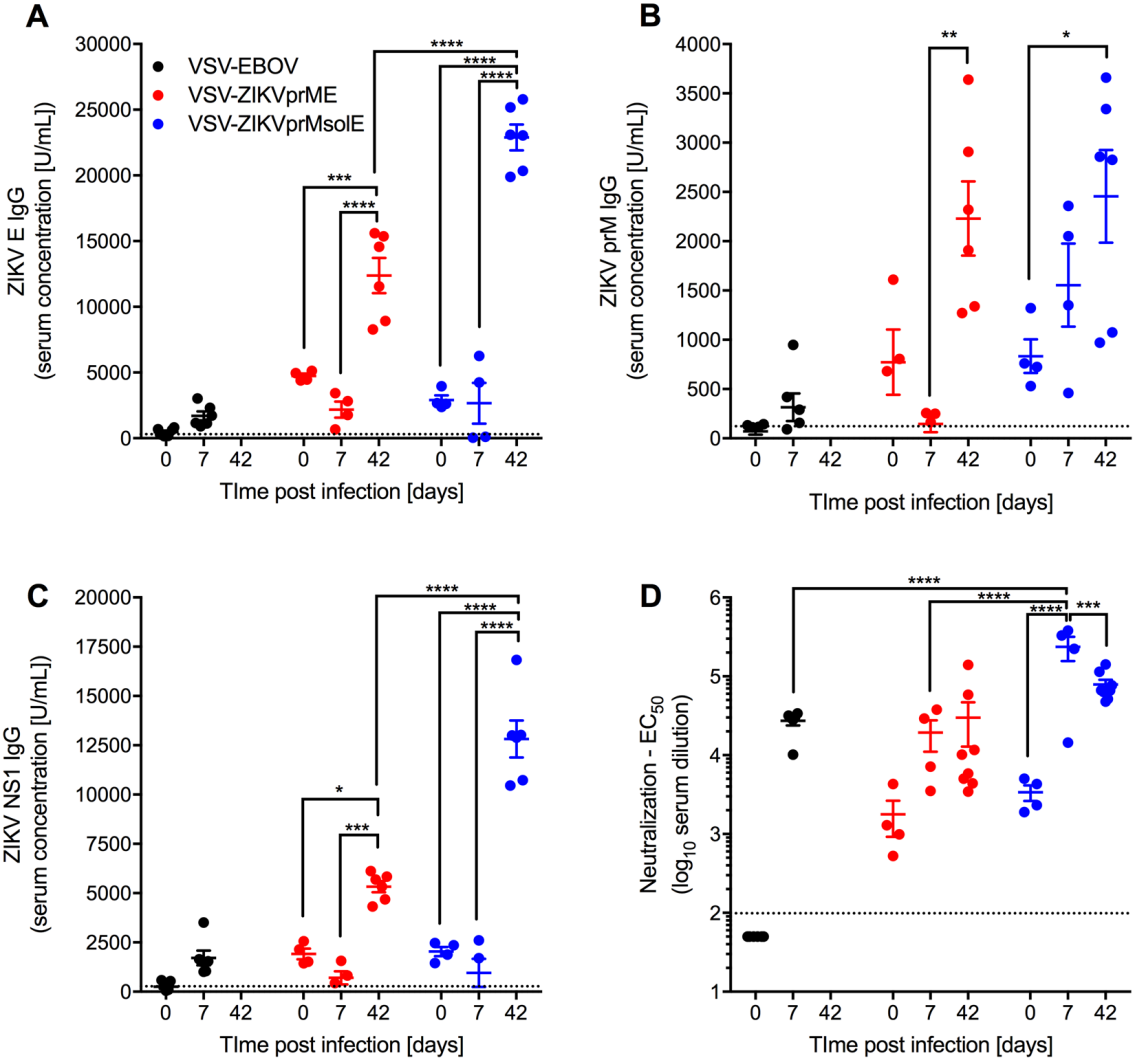
Humoral immune responses against ZIKV after vaccination and challenge. IFNAR^−/−^ mice were IM vaccinated with a single dose of vaccine 28 days prior to lethal ZIKV challenge. ELISA results depicting the levels of ZIKV antigen-specific IgG present in serum samples collected on day 0, 7 and 42 after lethal ZIKV challenge. (**A**) ZIKV E; (**B**) ZIKV prM; (**C**) ZIKV NS1. (**D**) ZIKV neutralizing activity of mouse serum samples as measured by the half maximal effective concentration (EC_50_). Statistically significant differences are indicated as follows: ****p < 0.0001, ***p < 0.001, **p < 0.01 and *p < 0.05.

Analysis of serum samples obtained from VSV-ZIKV vaccinated IFNAR^−/−^ mice on the day of challenge (n = 4) and on day 7 (n = 4) demonstrate the presence of antigen-specific antibodies (Fig. 3A,B). For all vaccinated animals the ZIKV E-specific IgG levels increased significantly between day 7 and day 42 (end of study) indicating an anamnestic antibody response suggestive of challenge virus replication (Fig. 3A). However, the effect is significantly more pronounced in the VSV-ZIKVprMsolE group compared to the VSV-ZIKVprME group suggestive of more challenge virus replication in the animals that received the ZIKVprMsolE vaccine (Fig. 3A). This is further supported by significantly higher amounts of ZIKV NS1 IgG in the survivors of the VSV-ZIKVprMsolE group compared to the VSV-ZIKVprME group (Fig. 3C) suggesting that the antigenicity of prME is superior to the prMsolE. Neutralizing antibodies against ZIKV were notably present in all VSV-ZIKV-vaccinated mice, but not in VSV-EBOV-vaccinated mice at the day of challenge. While control mice developed neutralizing antibodies by their terminal endpoint (day7, day 8), the vaccinated animals presented a slightly boosted ZIKV-specific neutralizing response by day 7 and at the end of the study (day 42)(Fig. 3D). On day 7, the neutralizing antibody titer in the VSV-ZIKVprMsolE group was significantly higher than in the VSV-ZIKVprME group, again suggesting that VSV-ZIKVprME limited viral replication to a greater degree (Fig. 3D).

### VSV-ZIKV vaccine efficacy against EBOV infection in CD1 mice

**Figure 4:**
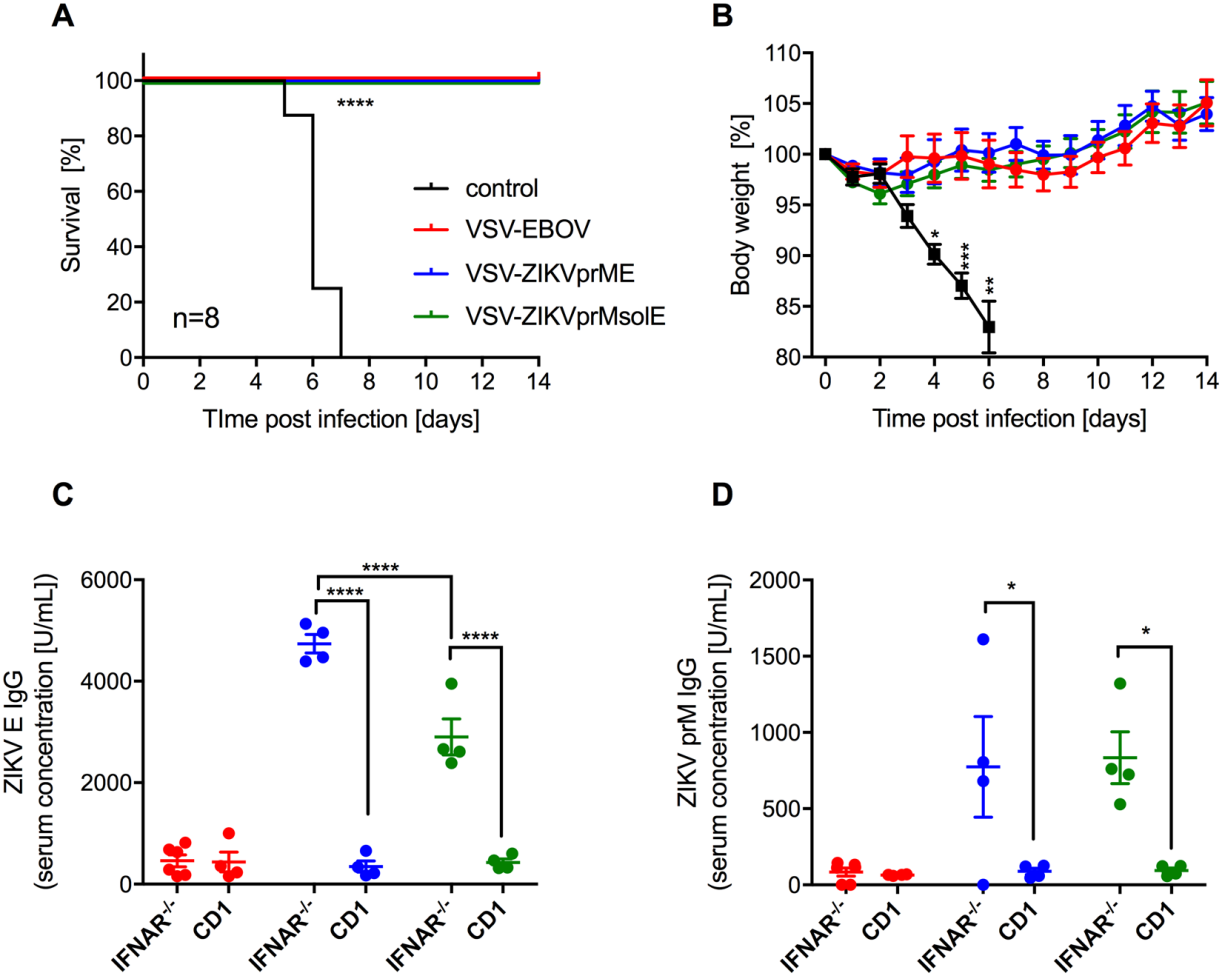
Efficacy of the VSV-ZIKV vaccines against lethal MA-EBOV challenge. Groups of CD1 mice were IM vaccinated with a single dose of vaccine 28 days prior to lethal MA-EBOV challenge. (**A**) Survival curves after lethal MA-EBOV challenge. (**B**) Body weight changes in the CD1 mice after MA-EBOV challenge. (**C**) ZIKV E- and (**D**) ZIKV prM-specific IgG responses were analyzed in the serum of IFNAR^−/−^ and CD1 mice (n = 4 each) 28 days after vaccination (time of challenge). Statistically significant differences are indicated as follows: ****p < 0.0001, ***p < 0.001, **p < 0.01 and *p < 0.05.

In the next experiment we sought to determine if the VSV-ZIKV vaccines, which are based on VSV-EBOV and seem further attenuated than the parental VSV-EBOV vaccine, still confer protection against lethal EBOV infection. Four groups of CD1 mice (n = 12) were IM vaccinated with 1 × 10^4^ pfu of either VSV-EBOV, VSV-ZIKVprME, VSV-ZIKVprMsolE, or remained unvaccinated. Challenge occurred 28 days later IP with 1,000 LD_50_ of mouse-adapted (MA)-EBOV for 8 animals per group, the other 4 mice were euthanized for serum collection. As expected, all control animals developed severe disease and met euthanasia criteria between days 5 and 7 post-challenge (Fig. 4A,B). In contrast, the three vaccines, VSV-EBOV and both VSV-ZIKV vaccines, uniformly protected CD1 mice against lethal disease (Fig. 4A). No outward signs of disease were observed, and weights gradually increased throughout the experiment (Fig. 4B) demonstrating that the addition of the second antigen upstream of EBOV GP in the VSV backbone does not compromise the protective efficacy against lethal MA-EBOV challenge.

Serum samples from the 4 mice per group euthanized 28 days post vaccination (day of challenge) were analyzed by ELISA. The ZIKV E- and ZIKV prM-specific IgG responses were compared to the ELISA data from the above described vaccinated IFNAR^−/−^ mice (Fig. 3A,B). For both vaccines, VSV-ZIKVprME and VSV-ZIKVprMsolE, the ZIKV antigen-specific immune responses were significantly higher in IFNAR^−/−^ mice compared to CD1 mice (Fig. 4C,D) suggesting a bias towards humoral immune responses in these immunocompromised mice.

### Time to immunity of the VSV-ZIKVprME vaccine in the IFNAR^−/−^ mouse model

**Figure 5:**
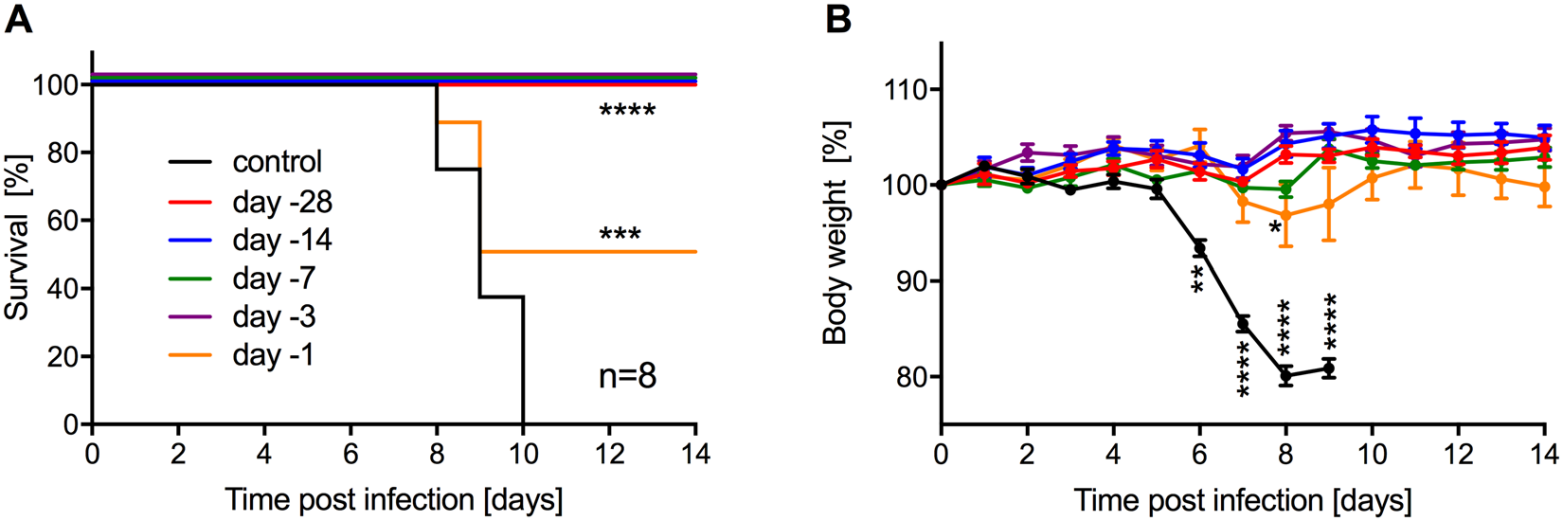
Time to immunity of the VSV-ZIKVprME vaccine against lethal ZIKV challenge. IFNAR^−/−^ mice were IM vaccinated with a single dose of VSV-ZIKVprME at the indicated time prior to lethal ZIKV challenge. Control mice received the same dose of the VSV-EBOV-Andes virus vaccine 28 days prior to ZIKV challenge. (**A**) Survival curves after lethal ZIKV challenge. (**B**) Body weight changes after ZIKV challenge. Statistically significant differences are indicated as follows: ****p < 0.0001, ***p < 0.001, **p < 0.01 and *p < 0.05.

As VSV-EBOV is a very fast-acting vaccine^27^, we analyzed the time to immunity of a single IM dose of 1 × 10^4^ pfu of the VSV-ZIKVprME vaccine, the superior vector, by vaccinating mice at various time points prior to lethal ZIKV challenge. Uniform protection from disease was observed when IFNAR^−/−^ mice were vaccinated at least 3 days prior to ZIKV challenge and 50% survival was observed when mice were vaccinated 1 day prior to challenge (Fig. 5A). In this group, 5 out of the 8 mice experienced weight loss around day 7 and 8, including three of the four mice that succumbed to disease. All other groups did not exhibit statistically significant changes in body weight over the course of the experiment and survived (Fig. 5B). These data demonstrate that despite the attenuation of the VSV-ZIKVprME vaccine compared to its parental vector VSV-EBOV, immunity is still conferred within 3 days.

## Discussion

The goal of vaccines is to maximize immunogenicity and protection while minimizing reactogenicity and potential adverse effects of vaccination in individuals. This often requires a delicate balance between potently stimulating the host immune response and avoiding damage to the host. The vaccines generated in this study, VSV-ZIKVprME and VSV-ZIKVprMsolE, appear to achieve such a balance. Although many pre-clinical ZIKV vaccine candidates have been developed recently^26,34,50–55^, the VSV-EBOV platform has several favorable advantages, including single-dose delivery, targeting of important immune cells, rapid time to immunity, and potent efficacy. The VSV-ZIKV vaccines generated here provided 100% protective efficacy against lethal ZIKV challenge in IFNAR^−/−^ mice.

The results showed that the VSV platform is an effective vaccine platform for the delivery and co-expression of the ZIKV and EBOV antigens, sufficient to control challenge virus replication and confer protection against lethal disease. Moreover, whereas VSV-EBOV caused disease in 10/16 IFNAR^−/−^ mice, the VSV-ZIKV vaccines were not associated with any adverse effects in these immunocompromised mice confirming further attenuation of these vaccine vectors. The *in vitro* growth kinetics reflect this finding, as both VSV-ZIKV vaccines grew slower and to a slightly lower, but not significantly different, peak titer than VSV-EBOV (Fig. 1B). Despite attenuation, the VSV-ZIKV vaccines still elicited protective antigen-specific humoral immune responses including ZIKV-neutralizing antibodies (Fig. 3). This echoes what has been reported for VSV-EBOV against EBOV challenge, which protects on the basis of a strong, adaptive, humoral response^56^. In terms of protection, both VSV-ZIKVprME and VSV-ZIKVprMsolE had comparable overall efficacy against ZIKV, demonstrating that that the C-terminal transmembrane region of the ZIKV E protein, and hence SVP formation, are not required for immunogenicity. Both VSV-ZIKV vaccines elicited similar levels of ZIKV-neutralizing activity, except on day 7 when the neutralizing activity of VSV-ZIKVprMsolE was significantly higher (Fig. 3D). This and the significantly higher levels of ZIKV E and NS1 IgG in the VSV-ZIKVprMsolE group at day 42 suggest that the full-length construct, VSV-ZIKVprME, might be better at controlling ZIKV replication.

ZIKV E and ZIKV prM-specific IgG responses in serum obtained 28 days after vaccination from IFNAR^−/−^ and CD1 mice were significantly higher in IFNAR^−/−^ mice compared to CD1 mice (Fig. 4C,D). This is not unexpected as VSV-based vaccines replicate efficiently in mice deficient of an innate immune response (e.g. IFNAR^−/−^ mice) compared to wildtype mice (e.g. CD1 mice)^57^. VSV is very sensitive to the host’s interferon response and, therefore, VSV replication is effectively controlled by the host’s innate immune system. Using IFNAR^−/−^ mice for efficacy testing of both VSV-ZIKV vaccines against lethal challenge was successful as VSV-based vaccines generally mediate protection via humoral immune responses^39^.

Adoptive transfer studies involving ZIKV challenge in mice have found that a high concentration ZIKV-specific IgG is sufficient to confer protection, even in the absence of CD4 and CD8 T cells^54^. In our study, VSV-ZIKV generated high titers of ZIKV-specific IgG, including uniformly higher concentrations of neutralizing antibodies pre-challenge compared to VSV-EBOV (Fig. 3D). Additionally, by day 42, all surviving IFNAR^−/−^ mice developed significantly increased concentrations of ZIKV E, prM, and NS1 IgG. For all of these reasons, we believe the VSV-ZIKV vaccines protect predominantly via a strong humoral response.

Similar to several previous VSV-based vaccines, VSV-ZIKVprME and VSV-ZIKVprMsolE are fast-acting and provide protection within three days of vaccination (Fig. 5). Although the precise mechanism of this protective activity is still poorly characterized, VSV-EBOV provides rapid protection against EBOV via involvement of the innate immune system combined with an early adaptive response^33^. We were surprised to see the rapid protection in the IFNAR^−/−^ mouse model as these mice are affected in their innate response capabilities. The mechanism of this rapid protection need to be further studied. Nevertheless, our studies further affirm the proposed use of the VSV platform for emergency vaccines that can be deployed to quickly contain outbreaks by using a ring vaccination approach^43^. This may be of special interest to geographically isolated regions with *Aedes* vectors, such as the Pacific islands, where containment might be most feasible during an outbreak. However, as a replicating vector the VSV might not be an ideal solution for vaccination of at risk groups like pregnant women during a public health emergency. Other vaccines might be more appropriate here as well as for population-based vaccination approaches, in particular the DNA vaccines that have recently been reported to be safe and immunogenic in a phase 1 human clinical trial^25^. However, if rapid immunity is needed in certain target groups such as women in the child-bearing age, VSV-based ZIKV vaccines might have a benefit even though proper risk assessment needs to be provided first.

It has already been demonstrated that VSV can be used to deliver multiple antigens and that recombinant VSVs can be pseudotyped with morphologically similar glycoproteins, like glycoproteins from the EBOV, Marburg virus and Nipah virus^30,35^. The expression of a flavivirus antigen, however, represents a new direction for this VSV-based vaccine vector. While the VSV and EBOV GP are timers, the ZIKV E protein encoded by our VSV vaccine vectors covers the ZIKV particle surface as a dimeric structure and matures through a different pathway in the cell. However, this protein appears to be properly expressed in vaccine-infected cells as shown by its immunogenicity, and expression is not essential for VSV replication as the EBOV GP is present. This work establishes a successful method for VSV-EBOV-based vaccination against ZIKV which may also be applicable to other flaviviruses. Both mosquito and tick-borne varieties of flaviviruses use a similar strategy of translocating their polyprotein into the endoplasmic reticulum membrane^58^. The complex formed by the prM-E proteins is critical to conformational maturation^24^. The inclusion of a Kozak sequence and JEV signal peptide prior to the ZIKV-antigen encoding sequences was evidently sufficient to ensure suitable transcription, translocation into the endoplasmic reticulum and translation. This opens the door for the creation of VSV-based vaccines against numerous other flaviviruses, including select agents such as Kyasanur Forest disease virus, the tick-borne encephalitis viruses, and Omsk hemorrhagic fever virus.

## Methods

### Ethics statement

All infectious *in vitro* work was performed in either a biosafety level 2 (BSL-2; for VSV and ZIKV) or BSL-4 (for MA-EBOV) laboratory setting at the Integrated Research Facility (IRF), Rocky Mountain Laboratories (RML), Division of Intramural Research (DIR), National Institute of Allergy and Infectious Disease (NIAID), National Institutes of Health (NIH) in Hamilton, MT, or at the Laboratory of Viral Diseases (DIR, NIAID, NIH) in Bethesda, MD. All infectious *in vivo* work was performed in either a BSL-2 or BSL-4 laboratory at RML and approved by the RML Institutional Animal Care and Use Committee (IACUC) and according to the guidelines of the Association for Assessment and Accreditation of Laboratory Animal Care, International (AAALAC) and our Office of Laboratory Animal Welfare (OLAW), assurance number (#A4149-01). All procedures were carried out by certified personnel. Humane endpoint criteria in compliance with IACUC-approved scoring parameters were used to determine when animals should be humanely euthanized.

### Generation of VSV-ZIKV constructs

Two codon-optimized ZIKV antigen-encoding sequences were selected, prME (nt 474–2491 of gi|1000381324|gb|KU681081.3|) and prMsolE (nt 474–2346 of gi|1000381324|gb|KU681081.3|). The soluble E (solE) antigen consists of the E protein deleted of its transmembrane domain. A Kozak sequence and a 15 aa JEV signal peptide-encoding sequence^59^ were appended immediately upstream to ensure proper antigen expression and translocation into the endoplasmic reticulum. These sequences were ordered from Eurofins Scientific (Brussels, Belgium) and cloned into the intergenic region between the first and second positions of the VSV-EBOV plasmid encoding the EBOV-Mayinga GP^38^, using PacI and AscI (NEB, Ipswich, MA) restriction sites^60^. Viral recovery was performed via co-transfection and blind passage of BHK-T7 cells onto VeroE6 cells as previously described^61^. Finally, the complete sequence of the recovered vaccine viruses was confirmed by Sanger sequencing.

### Cells and viruses

Vero and VeroE6 (both African green monkey kidney origin) cells were grown at 37 °C and 5% CO_2_ in Dulbecco’s modified Eagle’s medium (DMEM) (Sigma-Aldrich, St. Louis, MO) containing 2–10% fetal bovine serum (FBS) (Wisent Inc., St. Bruno, Canada), 2 mM L-glutamine (Thermo Fisher Scientific, Waltham, MA), 50 U/mL penicillin (Thermo Fisher Scientific), and 50 μg/mL streptomycin (Thermo Fisher Scientific). BHK-T7 (baby hamster kidney) cells were grown at 37 °C and 5% CO_2_ in minimum essential medium (MEM) (Thermo Fisher Scientific) containing 10% tryptose phosphate broth (Thermo Fisher Scientific), 2% FBS (Wisent), 2 mM L-glutamine (Thermo Fisher Scientific), 50 U/mL penicillin (Thermo Fisher Scientific), and 50 μg/mL streptomycin (Thermo Fisher Scientific). C6/36 (*Aedes albopictus*) cells were grown at 32 °C and 5% CO_2_ in MEM (Thermo Fisher Scientific) containing 10% FBS (Wisent), non-essential amino acids solution (Thermo Fisher Scientific), 50 U/mL penicillin (Thermo Fisher Scientific), and 50 μg/mL streptomycin (Thermo Fisher Scientific). The ZIKV used for challenge was the French Polynesian strain propagated on C6/36 cells^47^. Mouse-adapted EBOV was used for the challenge study in CD1 mice^62^. VSV-EBOV was used as a control vaccine^30^. The VSV-EBOV-Andes virus vaccine was used as the control vaccine in the time to immunity study^63^.

### Vaccine titration

VeroE6 cells were seeded in 6-well plates and grown to >95% confluency. VSV-ZIKV vaccines were serially diluted from 10^−2^ to 10^−9^ and used to infect wells in duplicate (0.5 ml each well). Plates were incubated for 1 hour at 37 °C with rocking, subsequently the inoculum was removed, and the cells were overlayed with 2 ml of a 1:1 mixture of 2% LMP agarose (Invitrogen, Carlsbad, CA) in 2 × MEM/2% FBS (Thermo Fisher Scientific). The agarose was allowed to solidify, and cells were incubated for ~72 hours 37 °C. Crystal violet solution staining was used to visualize plaques and the titer for each vaccine was calculated in pfu/ml.

### Growth kinetics

VeroE6 cells were grown to confluency in a 12-well plate and infected with a MOI of 0.01 with VSV wild-type, VSV-EBOV, VSV-ZIKVprME, and VSV-ZIKVprMsolE in triplicate. Inoculum was removed and replaced with DMEM/2% FBS. Supernatant samples were collected at 0, 6, 12, 24, 36, 48, 72, and 96 hours post-infection and stored at −80 °C until titration on VeroE6 cells.

### Immunofluorescence assay

VSV- and ZIKV-infected cells were fixed with 2% paraformaldehyde (PFA) 42 and 68 hours post-infection respectively, and permeabilized using 0.05% Triton X-100 in PBS. Blocking was performed for 1 hour at room temperature in PBS with 1% BSA. Primary staining was performed using anti-ZIKV E ZKA64 (Absolute Antibody, Oxford, UK), anti-VSV M 23H12 (Kerafast Inc., Boston, MA), and anti-EBOV GP 12/1.1 (kindly provided by Ayato Takada, Hokkaido University, Sapporo, Japan) antibodies. Secondary staining was performed using Alexa Fluor 488 goat anti-mouse IgG H + L (Invitrogen). Images were taken using 480 nm light on a ZOE fluorescent cell imager (Bio-Rad).

### Western blot analysis

VSV-infected cell supernatant samples were diluted 1:1 with 4× SDS buffer containing 20% β-mercaptoethanol and heated to 98 °C for 10 min. After loading onto a TGX criterion pre-cast gel (Bio-Rad Laboratories, Hercules, CA) in 10% sodium dodecyl sulfate-polyacrylamide gel electrophoresis loading buffer, proteins were separated by electrophoresis and transferred to a Trans-Blot polyvinylidene difluoride (PVDF) membrane (Bio-Rad). Following blocking overnight at 4 °C in PBS with 5% powdered milk and 0.05% Tween 20 (Fisher Scientific), protein detection was performed using the following mouse monoclonal antibodies: anti-ZIKV E 1176-56 (BioFront, Tallahassee, FL), anti EBOV-GP 12/1.1 (kindly provided by Ayato Takada, Hokkaido University, Sapporo, Japan), and anti-VSV M 23H12 (Kerafast Inc., Boston, MA). Secondary staining was performed using anti-mouse IgG (Jackson ImmunoResearch, West Grove, PA). Imaging was performed using SuperSignal West Pico chemiluminescent substrate (Thermo Scientific) and a FluorChem E system (ProteinSimple, San Jose, CA).

### Serial passaging

VeroE6 cells were infected with the VSV-ZIKV vectors in duplicate (MOI = 0.1) and the viruses were serially passaged by transferring 25% of the supernatant to freshly confluent VeroE6 cells every 48 hours (approximate MOI = 0.1). Supernatant was collected and RNA from each timepoint was extracted with a QIAmp viral RNA extraction kit (Qiagen, Germantown, MD). Primers flanking the PacI/AscI ZIKV antigen insertion site were used to amplify the VSV genome. Gel electrophoresis was performed in a 1% agarose gel in 1× tris-acetate-EDTA buffer and imaged using GelRed nucleic acid stain (Phenix Research, Candler, NC) and 302 nm UV. Western blot analysis was performed as described above.

### Mouse studies

Groups of 16 C57BL/6 IFNAR^−/−^ mice (male and female) were vaccinated IM with 1 × 10^4^ pfu in 0.1 ml (2 sites, 0.05 ml each) on day −28 with VSV-EBOV, VSV-ZIKVprME, or VSV-ZIKVprMsolE. On the day of challenge (day 0) four animals from each VSV-ZIKV group were euthanized for blood collection. The remaining 12 animals in the vaccinated groups were challenged IP with 5,000 pfu (1,000 LD_50_) of ZIKV^47^. Note, due to unexpected lethality, only 6 surviving VSV-EBOV mice were bled (cheek bleed), challenged and observed for signs of ZIKV infection. On day 7 post-challenge four animals from the VSV-ZIKV groups were euthanized for sample collection. Samples were also collected from the 6 control mice at the time of euthanasia. Surviving mice were monitored until 42 days post-infection when a single, terminal blood sample was collected.

Female CD1 mice were obtained from Envigo (Somerset, NJ). Groups of 12 mice were vaccinated IM with 1 × 10^4^ pfu in 0.1 ml (2 sites, 0.05 ml each) on day −28 with DMEM (control), VSV-EBOV, VSV-ZIKVprME, or VSV-ZIKVprMsolE. On day 0, 4 mice in each group were euthanized for serum collection and the remaining 8 mice per group were challenged IP with 1,000 LD_50_ of MA-EBOV^64^. Surviving mice were monitored until 42 days post-infection when a single, terminal blood sample was collected.

To evaluate the time to immunity of the VSV-ZIKVprME vaccine, five groups of 8 IFNAR^−/−^ mice (female and male) were IM vaccinated on day −28, day −14, day −7, day −3, or day −1 with 1 × 10^4^ pfu in 0.1 ml (2 sites, 0.05 ml each). On day −28, a single group of 12 mice (male and female) was vaccinated with the same dose and by the same route with 1 × 10^4^ pfu in 0.1 ml (2 sites, 0.05 ml each) of a control vaccine, the VSV-EBOV-Andes virus vaccine (VSV-ANDV, a previously generated VSV vector expressing ANDV GP and EBOV GP)^44^. All the groups were challenged IP with 5,000 pfu (1,000 LD_50_) of ZIKV and observed for signs of disease after infection. Surviving mice were monitored until 42 days post-infection when a single, terminal blood sample was collected.

### ZIKV titrations

Growth kinetic and mouse blood samples were thawed, and 10-fold serial dilutions were prepared. Tissue samples were homogenized by adding 1 ml of plain DMEM and a stainless-steel bead at 30 Hz for 10 min using a TissueLyser II (Qiagen). Tissue debris was sedimented at 8,000 rpm for 10 min, then 10-fold serial dilutions were prepared. All dilutions were inoculated onto confluent wells of VeroE6 cells in triplicate. The cytopathic effect was monitored until at least 96 hours post-inoculation and TCID_50_ was calculated for each sample employing the Reed and Muench method^65^.

### ELISA and neutralization assay

For enzyme linked immunosorbent assay (ELISA), the mouse serum samples were diluted 1:100 and assayed using the manufacturer’s protocol for ZIKV E-, prM-, and NS1-specific IgG (Alpha Diagnostic International).

The neutralization assay was performed as previously described^66^. In brief, serial 4-fold dilutions of heat-inactivated mouse serum samples were incubated with ZIKV H/PF/2013 reporter virus particles for 1 hour at 37 °C, followed by infection of pre-plated Vero cells. GFP-positive cells were quantitated by flow cytometry 2 days later. Titers with a half maximal effective concentration (EC_50_) were calculated by non-linear regression analysis of the dose-response curves. The initial dilution of sera was 1:100, which was set as the limit of detection for the assay. Values ≤ 100 were reported as a titer of 50. Titers shown represent the average of two independent experiments, each performed with technical replicates.

### Statistical analysis

All statistical analysis was performed in Prism 7 (GraphPad). The *in vitro* growth kinetics of recombinant VSV-ZIKVs (Fig. 1B), as well as the ELISA and EC_50_ data (Figs 3, 4C,D) were examined using one-way ANOVA with Tukey’s multiple comparisons to evaluate statistical significance at all timepoints. Animal body weight data were evaluated using the same method at critical timepoints (days 6–10 for ZIKV challenge studies; days 3–6 for MA-EBOV challenge study) (Figs 2B, 4B, 5B). Survival curves depicted in Figs 2A, 4A, 5A were examined for statistical significance using the Mantel-Cox test. Statistically significant differences are indicated as follows: ****p < 0.0001, ***p < 0.001, **p < 0.01 and *p < 0.05.

## Electronic supplementary material

Supplementary Dataset
