# Supplementary material for: A VSV-based Zika virus vaccine protects mice from lethal challenge
Source: Sci Rep. 2018 Jul 23;8:11043. doi: 10.1038/s41598-018-29401-x (PMC6056530; doi:10.1038/s41598-018-29401-x)
Supplement: Supplementary file 1 — Supplementary Dataset [file 41598_2018_29401_MOESM1_ESM.pdf]

A VSV-based Zika virus vaccine protects mice from lethal challenge

Jackson Emanuel, Julie Callison, Kimberly A. Dowd, Theodore C. Pierson, Heinz Feldmann, and Andrea Marzi

Supplementary information

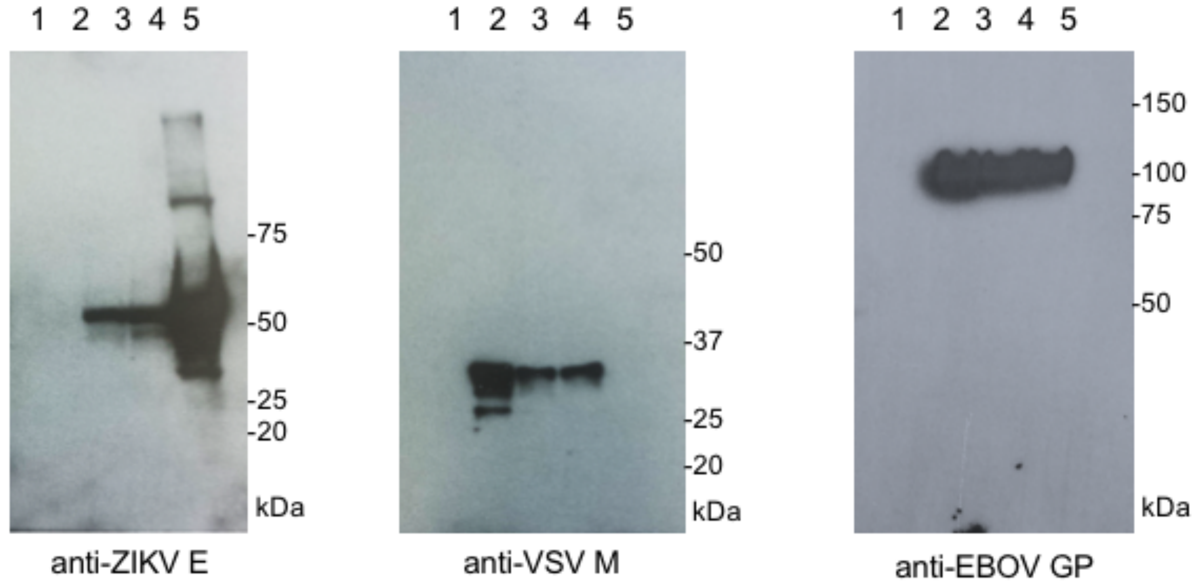

Figure S1. VSV-ZIKV vaccine protein expression. VeroE6 cells were infected with ZIKV or VSV vaccines and the supernatant was collected. Western blot analysis confirmed the presence of the viral proteins present in the supernatant of VSV- or ZIKV-infected cells. Lane 1, uninfected control; lane 2, VSV-EBOV; lane 3, VSV-ZIKVprME; lane 4, VSV-ZIKVprMsolE; lane 5, ZIKV.

A

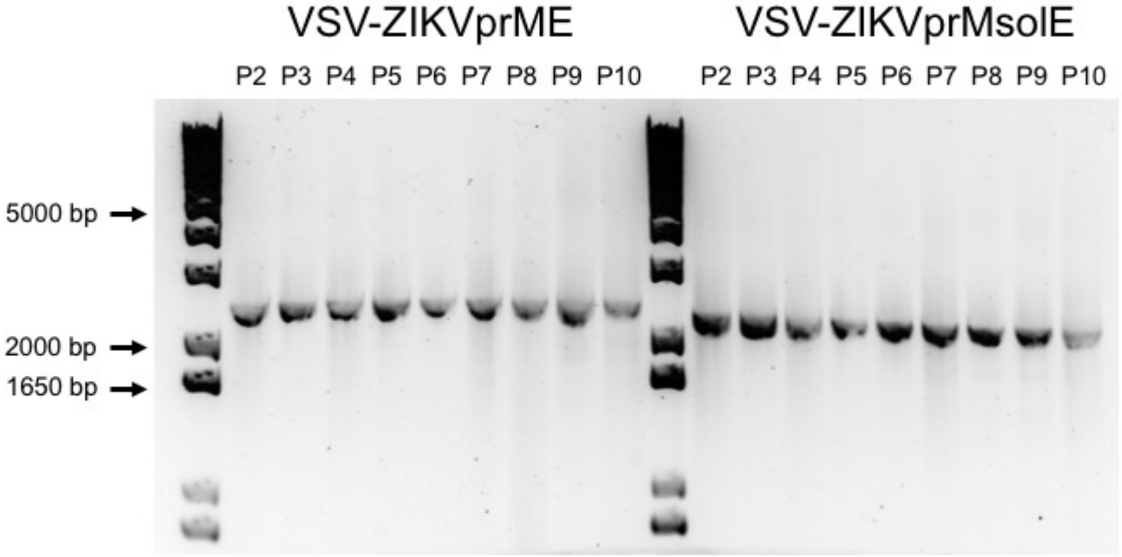

B

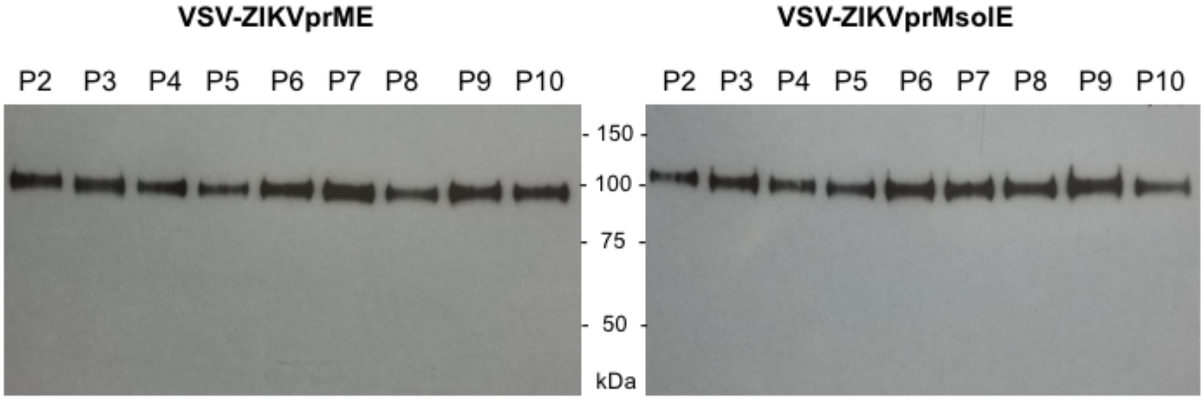

Figure S2. Maintenance of the ZIKV antigen-encoding sequence in the recombinant VSV-ZIKV genomes. VSV-ZIKV vectors were serially passaged 10 times and supernatant samples were collect for antigen verification. **(A)** The PCR products of the ZIKV antigen-encoding region of recombinant VSV-ZIKV genome on one agarose gel are shown. Labels indicate the successive passage numbers from passage 2 through 10. The expected band sizes are ~2.0 kb (for VSV-ZIKVprME) and ~1.9 kb (for VSV-ZIKVprMsolE). **(B)** Western Blot analysis was performed to confirm EBOV GP expression in the supernatant of the infected cells.
